# Exploring spatial patterns of vulnerability using linked health data

**DOI:** 10.23889/ijpds.v8i3.2280

**Published:** 2023-09-11

**Authors:** Abigail Brake, Daniel Birks, Mark Mon-Williams, Sam Relins

**Affiliations:** 1 University of Leeds; 2 Bradford Institute for Health Research

## Introduction & Background

The types of challenges police and ambulance services deal with often overlap, for instance supporting those who suffer from mental ill-health. Research has shown that emergency service problems often concentrate, but also that some individuals who come to the attention of one service may not be as visible to another despite their overlap in roles.

## Objectives & Approach

This study explored how routinely collected 999 data may reveal insights into how these services support potentially vulnerable populations. We argue that better understanding the nature and distribution of vulnerability-related calls may help to inform future preventative or harm reduction-based interventions. We analysed administrative data provided by Yorkshire Ambulance Service for the Bradford region through the Connected Bradford research database, posing the following questions: (1) can 999 call data provide insights into vulnerability-related incidents attended by ambulances?; (2) where and when are these incidents most prevalent?; and (3) what are the spatial patterns of calls and patient home locations associated with them?

## Relevance to Digital Footprints

We first select calls associated with nine callout reasons indicative of vulnerability. Patients can choose to share their data with each healthcare service they use, so we harnessed this digital footprint to analyse the spatial distribution of call locations (at postcode sector level) and patient home location (at MSOA level).

## Results

Results indicate substantial concentrations of vulnerability-related calls in multiple postcode sectors including the City Centre (where we estimate 18% of calls may be vulnerability-related) and several other areas which are associated with deprivation. Exploring flows of people from their home location to incident location we also see substantial spatial variation in the locations in which patients involved in these types of incidents reside.

## Conclusions & Implications

These analyses represent initial efforts to better understand how vulnerable groups are supported by public services, and have the potential to inform future resource allocation and targeting of upstream interventions.

